# Atomoxetine on neurogenic orthostatic hypotension: a randomized, double-blind, placebo-controlled crossover trial

**DOI:** 10.1007/s10286-024-01051-2

**Published:** 2024-09-19

**Authors:** Naome Mwesigwa, Patricio Millar Vernetti, Annet Kirabo, Bonnie Black, Tan Ding, Jose Martinez, Jose-Alberto Palma, Italo Biaggioni, Horacio Kaufmann, Cyndya A. Shibao

**Affiliations:** 1 Division of Clinical Pharmacology, Department of Medicine, Vanderbilt University Medical Center, 506 Robinson Research Building, Nashville, TN 37232-8802 USA; 2 Department of Neurology, New York University Grossman School of Medicine, New York, NY USA

**Keywords:** Autonomic failure, Norepinephrine transporter blocker, Norepinephrine reuptake inhibitor, Syncope, Dysautonomia

## Abstract

**Purpose:**

We previously reported that single doses of the norepinephrine transporter inhibitor, atomoxetine, increased standing blood pressure (BP) and ameliorated symptoms in patients with neurogenic orthostatic hypotension (nOH). We aimed to evaluate the effect of atomoxetine over four weeks in patients with nOH.

**Methods:**

A randomized, double-blind, placebo-controlled crossover clinical trial between July 2016 and May 2021 was carried out with an initial open-label, single-dose phase (10 or 18 mg atomoxetine), followed by a 1-week wash-out, and a subsequent double-blind 4-week treatment sequence (period 1: atomoxetine followed by placebo) or vice versa (period 2). The trial included a 2-week wash-out period. The primary endpoint was symptoms of nOH as measured by the orthostatic hypotension questionnaire (OHQ) assessed at 2 weeks.

**Results:**

A total of 68 patients were screened, 40 were randomized, and 37 completed the study. We found no differences in the OHQ composite score between atomoxetine and placebo at 2 weeks (−0.3 ± 1.7 versus −0.4 ± 1.5; *P* = 0.806) and 4 weeks (−0.6 ± 2.4 versus −0.5 ± 1.6; *P* = 0.251). There were no differences either in the OHSA scores at 2 weeks (3 ± 1.9 versus 4 ± 2.1; *P* = 0.062) and at 4 weeks (3 ± 2.2 versus 3 ± 2.0; *P* = 1.000) or in the OH daily activity scores (OHDAS) at 2 weeks (4 ± 3.0 versus 5 ± 3.1, *P* = 0.102) and 4 weeks (4 ± 3.0 versus 4 ± 2.7, *P* = 0.095). Atomoxetine was well-tolerated.

**Conclusions:**

While previous evidence suggested that acute doses of atomoxetine might be efficacious in treating nOH; results of this clinical trial indicated that it was not superior to placebo to ameliorate symptoms of nOH.

**Trial registration:**

ClinicalTrials.gov; NCT02316821.

**Supplementary Information:**

The online version contains supplementary material available at 10.1007/s10286-024-01051-2.

## Introduction

Neurogenic orthostatic hypotension (nOH) is defined as a fall in systolic blood pressure (SBP) of at least 20 mm Hg or diastolic blood pressure (DBP) of at least 10 mm Hg within 3 min of standing or head-up tilt [3–5], due to baroreflex dysfunction resulting in inappropriate release of norepinephrine in the neurovascular junction upon standing [11]. When the drop in BP upon standing is sufficient to cause organ hypoperfusion, patients with nOH experience symptoms including but not limited to lightheadedness, dizziness, falls, and syncope [1, 17]. nOH is a nonmotor feature of synucleinopathies, including Parkinson’s disease (PD), multiple system atrophy (MSA), dementia with Lewy bodies (DLB), and pure autonomic failure (PAF). These are a group of neurodegenerative diseases characterized by the intracellular accumulation of the misfolded protein α-synuclein in neurons and glia [7, 18].

The treatment of symptomatic nOH is challenging because of limited therapeutic options. Nonpharmacological treatments include water boluses, abdominal binders, and a high-salt diet, but these measures have limited efficacy. Only two drugs have been specifically approved by the US food and drug administration (FDA) for nOH and related symptoms: midodrine, an alpha-1 adrenergic agonist, and droxidopa, a synthetic norepinephrine precursor [22]. Other medications, such as fludrocortisone and pyridostigmine [24], are used off-label with limited success. Many patients fail to improve or develop intolerable side effects with these therapies. Thus, identifying effective therapies for symptomatic nOH is an unmet medical need.

Atomoxetine is FDA-approved for the treatment of attention deficit hyperactivity disorder [25–27]. It selectively blocks the norepinephrine transporter receptor in prejunctional neurons, increasing norepinephrine bioavailability in post-junctional alpha-1 adrenergic receptors and enhancing vasoconstriction. We previously reported that single low doses of up to 18 mg of atomoxetine significantly increased standing BP in patients with nOH and normal or elevated plasma norepinephrine levels (i.e., nOH caused by MSA) [19], compared with those with low plasma norepinephrine levels. Further, we reported that a single atomoxetine dose resulted in higher standing BP increases and more pronounced symptomatic reductions as measured by the orthostatic hypotension symptom assessment (OHSA) versus midodrine. Moreover, higher levels of plasma supine norepinephrine predicted the response to atomoxetine as measured by the increase in standing BP and the improvement in symptoms of nOH [23]. Collectively, this evidence suggested that inhibiting the norepinephrine transporter receptor with atomoxetine could be a potential therapeutic strategy for patients with nOH.

This early evidence was mostly gathered in studies using single low doses of atomoxetine. We hypothesized that a longer-term course with atomoxetine at similar low doses would have similar beneficial effects on symptoms of nOH. To test this hypothesis, we performed a clinical trial testing the effect of a 4-week course of atomoxetine on symptoms of nOH.

## Methods

### Trial design and participants

This was an oligo center, randomized, double-blind, placebo-controlled, crossover clinical trial, enrolling participants with nOH at two academic centers in the USA (Vanderbilt University, Nashville, TN and New York University Grossmann School of Medicine, New York, NY). The Multiple System Atrophy (MSA) Coalition, a patient advocacy group, collaborated by referring potential participants to the study sites. The study was registered at ClinicalTrials.gov (NCT02784535).

The trial enrolled nOH patients between the ages of 40 and 80 years old, and the diagnosis of nOH was ascertained according to published criteria [5]. Autonomic function tests were performed in all subjects to confirm autonomic failure. MSA was defined by the American Autonomic Society and the American Academy of Neurology [6].

Participants were excluded if they had a history of hypersensitivity to atomoxetine or other norepinephrine transporter inhibitors, previous history or current use of monoamine oxidase inhibitors, and concomitant use of strong CYP2D6 inhibitors. Participants with preexisting sustained hypertension [blood pressure (BP) ≥ 140/80 mmHg in the sitting position], impaired hepatic function, impaired renal function, and subacute cardiovascular disease defined as myocardial infarction within 6 months before enrollment, congestive heart failure [left ventricular (LV) hypertrophy acceptable], and history of serious neurologic disease (such as cerebral hemorrhage, stroke) were also excluded. Participants were permitted to continue fludrocortisone but all other pressor medications (including midodrine, droxidopa, atomoxetine, yohimbine, and pyridostigmine) were withdrawn for five half-lives before the administration of the study drug. All other medications, including fludrocortisone, were held constant throughout the study.

After obtaining informed consent, each participant underwent a complete medical history, physical examination, and routine safety laboratory analysis, including electrolytes and serum creatinine, blood count, metabolic panel, urinalysis, and electrocardiogram (EKG).

### Randomization and masking

Patients were randomized according to a computer-generated schedule, allocated to one of two treatment sequences (atomoxetine/placebo) or vice versa, and received a 4-week treatment, then crossed over for another 4 weeks after a 1-week washout period. The treatment assignment was stratified by clinical center and atomoxetine dose (10 or 18 mg twice a day). The study personnel and the participants were blinded.

The study drug was atomoxetine capsules, with 10 or 18 mg doses given twice daily (morning and afternoon) for 4 weeks. The manufacturer of the active ingredient of the study medication (atomoxetine) was Eli Lilly (branded name Strattera®) and a generic brand when available (NorthStar and Glenmark Pharmaceuticals). These companies did not sponsor, design, analyze, or fund the study. The active ingredient was over-encapsulated to maintain the blindness of the study. Placebo ingredients included blue opaque “oo” capsules (Letco item: 69,036) and microcrystalline cellulose to cover.

### Procedures

Before randomization, the study included an open-label phase (up to 2 days) to identify responders. In this phase, patients were initially administered a single dose of 10 mg of atomoxetine. If there was no response, they were asked to return the next day to receive an 18 mg dose. Responders were defined as those who: (i) experienced an improvement (decrease) of at least 1 point in the OHSA Question 1 (dizziness, lightheadedness, feeling faint, or feeling like you might black out) and (ii) had an increase in standing SBP of at least 10 mmHg from baseline 60-min after the single dose administration. BP and heart rate measurements were taken in the supine position every 5 min for 30 min during baseline, and every 5 min for 60 min after drug administration using the Omron Hem 907 × L IntelliSense Professional Digital Blood Pressure Monitor. BP and heart rate were also obtained while standing at 1, 3, 5, and 10 min or until tolerated. Participants were withdrawn if they did not respond to 18 mg of atomoxetine, or if they had sustained systolic BP (SBP) > 180 mm Hg or diastolic (DBP) > 110 mmHg diastolic while standing, sitting, or supine after two consecutive BP measures taken 15 min apart.

**Fig. 1:**
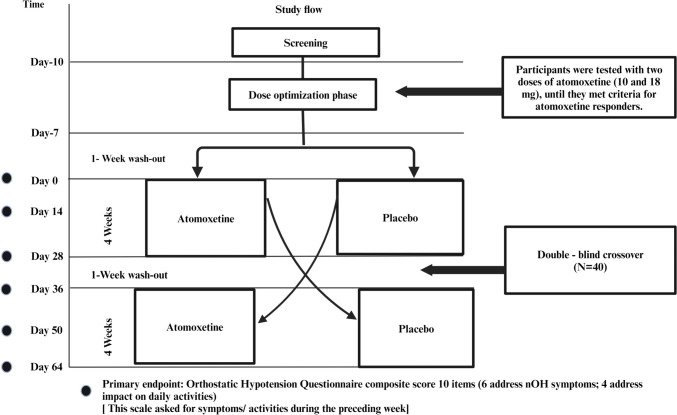
Study design. After optimizing the dose, there was a 1-week break before randomizing the subject to the first 4-week period. Another 1-week break was given before crossing over to the final 4-week period

Responders were subsequently randomized to receive the dose of atomoxetine they responded to (10 mg or 18 mg) twice daily (in the morning and the afternoon) or placebo for 4 weeks (period 1). Participants were then instructed to discontinue the study drug for 1 week (wash-out period), after which they were crossed over to the converse allocation in period 2 (placebo or atomoxetine) for an additional 4 weeks (Fig. 1).

Participants were closely monitored in each period by telephone visits on days 1, 3, 5, and 7 during the first week of each period followed by weekly telephone visits; these visits included assessment of orthostatic vital signs, study medication compliance, and evaluation of adverse events. Primary and secondary endpoints were measured twice; at 2 weeks (days 14 and 50) and 4 weeks (days 28 and 64) for each period.

The primary endpoint was the change from the baseline of the OHQ and the composite score at 2 weeks. Symptoms of nOH were assessed with the orthostatic hypotension questionnaire (OHQ) [9], a patient-reported Likert scale (0 represents no symptoms, 10 represents severe symptoms), whereas the secondary endpoint included the change in standing SBP, 60 min post-drug administration compared with the baseline.

### Data management and safety monitoring

Research personnel entered clinical data, laboratory test results, and research laboratory data into the Vanderbilt Redcap [14]. Before data was unblinded, the primary investigator (PI) and the study statistician reviewed all protocol deviations and determined a list of protocol violators to be removed for the per-protocol analysis. Safety endpoints such as adverse events were reported by the data and safety monitoring board (DSMB), which provided summary reports to the IRB, FDA, and the investigators. They met at least three times, once to review and ratify its charter and twice to receive reports on the progress of the study.

### Statistical analyses

In a previous study [20], the active treatment group had a mean and standard deviation (SD) change of −2 (2.1) units in OHQ composite score versus −0.9 (91.7) in the placebo treatment group. We assumed a similar effect was achieved by atomoxetine, a sample size of 40 gave 90% power to detect a difference in means of −0.9 (−0.83 versus. −0.93), with a standard deviation of 1.7 for the within-subject differences using a paired *t*-test at 0.05 type 1 error rate. For our secondary endpoint, the increase in standing SBP, 60 min post-drug administration, our sample size had > 90% power to detect a difference of 45 ± 23 mmHg between placebo and atomoxetine. Only participants who completed the full protocol were included in the final analysis. The study biostatistician calculated within-subject mean differences and 95% confidence intervals for the atomoxetine versus placebo comparison and tested for treatment effect using a paired *t*-test. Additional adjusted analyses were conducted using mixed effect models with a random subject effect and with treatment (atomoxetine versus placebo) as the fixed effect including age and gender as covariates in the model. We employed standard graphing and screening techniques to detect outliers; we did not find outliers and no data was removed. The data was assessed for normality. If normality was violated, we employed non-parametric methods of analysis. We analyzed the data using R version 4.2.0 software and expressed it as mean (SD) throughout the manuscript. Spearman’s rank correlation was used to assess the associations of continuous variables of interest using GraphPad Prism (version 9.4.0). Trend lines and confidence intervals (CI) were estimated with linear regression. *P* value < 0.05 was considered statistically significant.

## Results

**Fig. 2:**
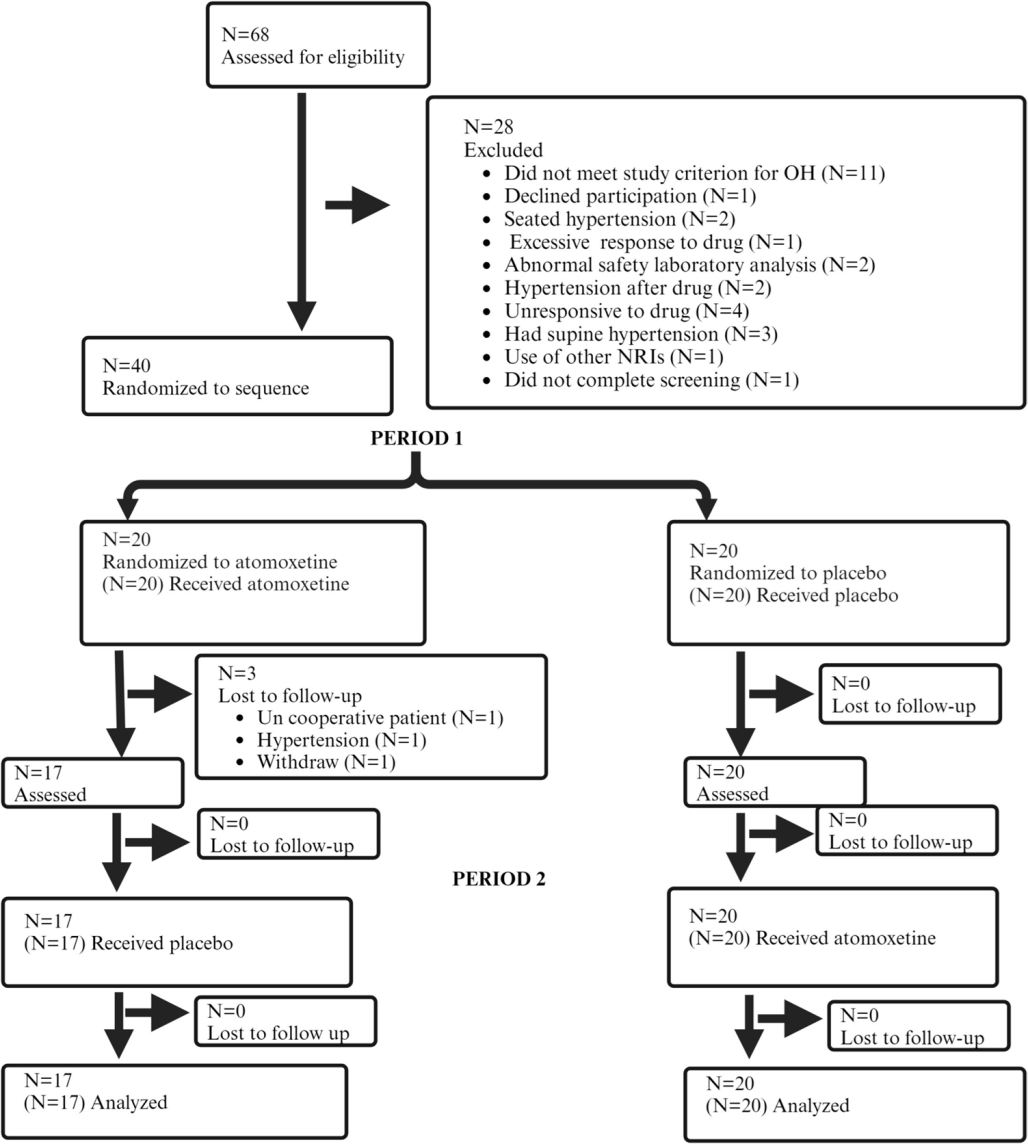
Consort enrollment flow diagram. The study design was a randomized, placebo-controlled, 2 × 2 crossover clinical trial. A total of 68 patients were assessed for eligibility, and 40 were randomized to two study sequences (atomoxetine-placebo or vice versa). Thirty-seven patients completed the trial

Between July 2016 and May 2021, we screened 68 participants, and all underwent the open-label, single-dose administration of atomoxetine. Of those, 40 responded and were allocated to treatment, and 37 patients completed the trial and were included in the final analysis per protocol (Fig. 2).

Demographic and clinical characteristics of participants are presented in Table 1. All the study subjects were non-Hispanic, mostly men (60%), average age of 68 ± 8.1 years, 41% MSA, 19% Parkinson’s disease, and 41% PAF.

**Table 1:** Descriptive characteristics of the study population

Characteristics	Placebo–atomoxetine (*n* = 20)	Atomoxetine–placebo (*n* = 20)	Total (*n* = 40)
Age (years)	68.4 (8.0)	67.3 (8.2)	67.9 (8.1)
Male	55%	65%	60%
Female	45%	35%	40%
Weight (kg)	77.0 (14.0)	84.0 (21.0)	80.0 (18)
Height (cm)	172.1 (9.6)	177.4 (9.8)	174.7(9.9)
BMI (kg/m^2^)	26.1 (4.7)	26.2 (5.0)	26.2 (4.8)

Data presented as mean (standard deviation), and body mass index (BMI)

### Outcomes and estimations

**Fig. 3:**
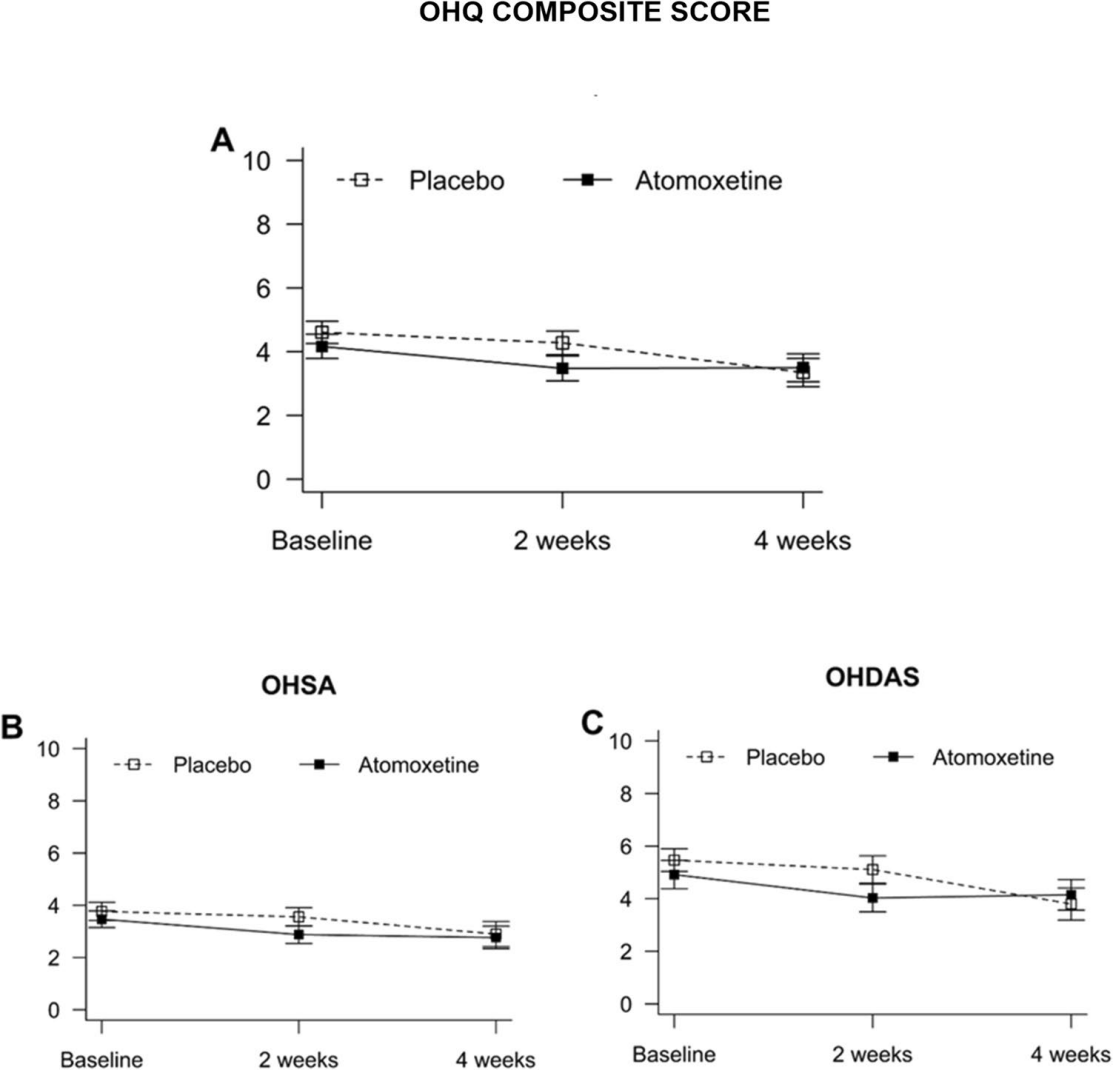
The effect of atomoxetine on symptoms related to neurogenic orthostatic hypotension. The changes in the orthostatic hypotension questionnaire composite score (OHQ composite score, Panel A), changes in orthostatic hypotension symptoms assessment (OHSA, Panel B), and changes in orthostatic hypotension daily activity score (OHDAS, Panel C) were evaluated. There were no significant changes between interventions during the 2- or 4-week treatment period. The data is presented as the mean and standard error of the mean. Higher symptom assessment scores indicate worse orthostatic symptoms

Treatment with 10–18 mg of atomoxetine did not significantly decrease NOH-related symptoms compared with the placebo. The primary endpoint, OHQ composite score, was similar between treatment groups (Fig. 3A). There were no significant differences in OHSA (Fig. 3B), and OHDAS (Fig. 3C) at 2 and 4 weeks during the study intervention. A subanalysis of each component of the OHSA and OHDAS found a significant decrease in fatigue at 2 weeks only (4 ± 2.5 versus 5 ± 2.8, *P* = 0.040) with atomoxetine.

**Fig. 4:**
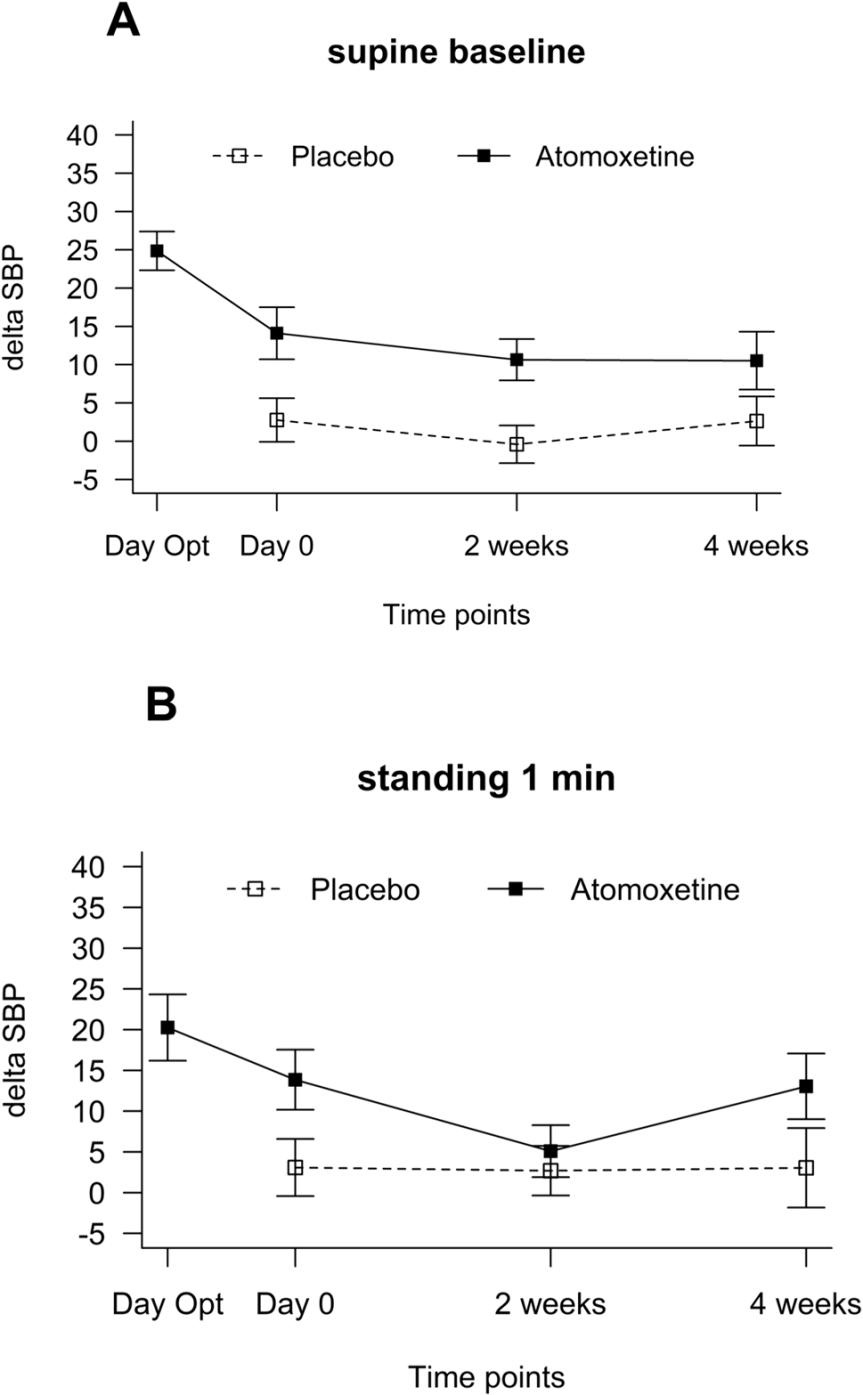
Changes in systolic blood pressure with atomoxetine and placebo. Systolic blood pressure (SBP) changes were monitored during the optimization phase (day opt.), at baseline (no intervention), and 2- and 4-weeks during atomoxetine versus placebo treatment periods in the supine position (**A**), and after 1 min of standing (**B**). During atomoxetine treatment, there was a decrease in supine and upright systolic blood pressure (SBP). The data is presented as the mean and standard error of the mean

During the optimization phase, 11 responded to 10 mg and 38 responded to 18 mg atomoxetine; atomoxetine significantly improved standing BP acutely (20 ± 22.6) and an an observed increase in supine SBP with atomoxetine during the optimization day was 25 ± 14.5 mmHg. However, this pressor response significantly declined from baseline, 2 weeks, and 4 weeks (Fig. 4). Compared with placebo, the pressor response to atomoxetine remained significant at 2 weeks only (11 ± 15.5 versus −0.4 ± 13.9 placebo, *P* = 0.04), but not at 4 weeks (11 ± 18.1 atomoxetine versus 3 ± 14.7 placebo, *P* = 0.120). Similarly, there was no significant difference in the change in standing SBP at 1 min at 2 weeks (5 ± 18.0 versus. 3 ± 16.7 placebo, *P* = 0.589), and 4 weeks (13 ± 19.3 versus. 3 ± 22.3 placebo, *P* = 0.122) between interventions.

Among the 40 subjects randomized in our study, 15 were diagnosed with central (MSA) autonomic failure versus 22 were diagnosed with peripheral autonomic failure. We performed a subanalysis based on MSA diagnosis and found no differences between atomoxetine and placebo in the OHQ composite score in the patients with MSA (−0.2 ± 1.2 versus −0.5 ± 1.9, *P* = 0.731) at 2 weeks post-treatment.

### Safety and tolerability

Safety was assessed by the adverse event (AE) rate. A total of 50 adverse effects were recorded; of these, 2 occurred during the dose optimization period, 28 occurred during atomoxetine, and 20 occurred on placebo. The 10 most common AE and those occurring more than once are listed in Supplementary Table 2. Three serious AE cases were reported, and two of the patients were in the atomoxetine group; one had a fever of unknown origin associated with disorientation and confusion, and the other had trouble swallowing. No death was reported during the trial.

**Table 2:** Primary endpoint; orthostatic hypotension questionnaire composite score

	Atomoxetine	Placebo	Difference	*P* value
OHQ composite score				
OHQ comp score baseline	4.2 (2.3)	4.6 (2.2)	0.3(1.8)	0.340
OHQ comp score 2 weeks	3.5 (2.2)	4.3 (2.2)	0.7 (2.1)	1.104
OHQ comp score 4 weeks	3.5 (2.3)	3.4 (2.0)	0.6 (1.3)	1.116
OHSA composite score				
OHSA comp score baseline	3.5 (1.9)	3.8 (2.2)	0.2 (1.9)	0.628
OHSA comp score 2 weeks	2.9 (2.0)	3.6 (2.1)	− 0.6 (1.8)	0.062
OHSA comp score 4 weeks	2.8 (2.2)	2.9 (2.2)	0.0 (1.0)	1.000
OHDAS composite score				
OHDAS composite score baseline	4.9 (3.3)	5.5 (2.6)	−0.5 (1.9)	0.137
OHDAS composite score 2 weeks	4.0 (3.0)	5.1 (3.1)	−0.9(2.8)	0.102
OHDAS composite score 4 weeks	4.2 (3.0)	3.8 (2.7)	0.9 (1.7)	0.095

Changes in orthostatic hypotension questionnaire (OHQ) and its components: orthostatic hypotension symptom assessment (OHSA) and orthostatic hypotension daily activity score (OHDAS) during treatment periods

## Discussion

Contrary to our hypothesis, continuing treatment with atomoxetine did not improve symptoms associated with nOH as measured by the OHQ composite score. Further, we observed a decline in the initial standing pressor response to atomoxetine over time, highly suspicious of tachyphylaxis, which could explain the lack of symptomatic benefit.

Several factors could explain our findings, atomoxetine increases norepinephrine concentration by blocking its reuptake, hence prolonging its effect on the postjunctional α1-adrenergic receptor to induce vasoconstriction. Therefore, eliciting an adequate pressor response in patients with nOH requires residual preservation of endogenous norepinephrine secretion and some degree of postjunctional α1-adrenergic hypersensitivity [23]. In our proof-of-concept study, we observed a substantial increase in blood pressure and improvement in symptoms in patients with MSA who have preservation of norepinephrine (NE) secretion. Considering that in nOH there are different degrees of post-ganglionic denervation, we enriched our study population and enrolled only patients who responded to an acute dose of atomoxetine. Unfortunately, our data showed that this initial response is not maintained over time, hence affecting our ability to demonstrate a symptomatic improvement with repetitive doses of atomoxetine. We performed a subanalysis based on the diagnosis of MSA (central autonomic failure), as ascertained by reviewing the patient’s history, neurological exam, and imaging. We found no differences in OHQ symptom assessment scores in MSA versus patients with PD and PAF, but our study was not powered to find differences based on patients’ diagnoses. Therefore, a larger study would be required to exclude a preferential symptomatic effect in MSA.

We selected pediatric dosages of atomoxetine (10 or 18 mg twice a day); it could be possible that the lack of efficacy with repetitive doses of atomoxetine is due, in part, to receptor desensitization or changes in the binding capacity in the prejunctional norepinephrine transporter (NET) receptors or the post-junctional alpha-1 adrenergic receptors. Tachyphylaxis is a common phenomenon in the treatment of depression with serotonin reuptake inhibitors [8]. In these cases, a drug “holiday” or completely stopping the medication and/or reducing the frequency of atomoxetine may help prevent this phenomenon.

Previous studies using a selective, long-acting norepinephrine reuptake inhibitor for symptomatic nOH ampreloxetine, reported a persistent effect on blood pressure and symptoms with continuous use over 20 weeks, associated with increased NE levels, and it was lost after drug discontinuation [10, 13]. This data indicated that different norepinephrine reuptake inhibitors (NRIs) may have distinctive durations of action. Importantly, atomoxetine has been associated with low NET occupancy rates, which are exponentially linked to the dosage administered [2, 21]. Thus, the low occupancy rate associated with the pediatric doses of atomoxetine may have negatively impacted our ability to detect an effect on nOH symptoms. Alternatively, designing a study that includes a dose-titration regimen of up to 40 mg (the atomoxetine adult dose) during the trial while monitoring for a significant increase in blood pressure may overcome the low occupancy rate and prevent the progressive decline in response.

Increasing the endogenous pool of norepinephrine could be another way to preserve the pressor and clinical response to atomoxetine in patients with autonomic failure. For example, atomoxetine could be an adjunct therapy to droxidopa, which works by increasing NE production in both neurons and nonneuronal cells that express l-aromatic amino acid decarboxylase and is available for treating nOH. Case reports in patients with refractory hypotension found a significant pressor effect with the coadministration of these two agents [12]. Likewise, the combination of pyridostigmine, a cholinesterase inhibitor that enhances cholinergic neurotransmission in sympathetic and parasympathetic ganglia, with atomoxetine appears to synergistically increase blood pressure and improve orthostatic tolerance [16]. Similarly, yohimbine improves the pressor effect of atomoxetine when given in combination in certain populations [15].

In conclusion, chronic treatment with 10 to 18 mg of atomoxetine did not improve nOH-related symptoms. This could be explained by the development of tachyphylaxis given the gradual decline in atomoxetine’s pressor effect. Further studies would be needed to determine if drug holidays, use of higher doses, or combination therapy with other drugs can improve the efficacy of atomoxetine in the management of nOH.

## Supplementary Information

Below is the link to the electronic supplementary material.

Supplementary file 1 : Linear regression analysis on the atomoxetine group. The relationship is not linear. Results of the Shapiro–Wilk test (*P* < 0.05) and Q–Q plots show none of the norepinephrine supine 15 min, dif_ohq_comp_score_2w, and dif_ohq_comp_score_4w in a normal distribution (TIF 1887 KB)

Supplementary file 2 (DOCX 17 KB)

Supplementary file 3 (DOCX 14 KB)

Supplementary file 4 (DOCX 20 KB)

Supplementary file 5 (DOCX 21 KB)
