# Supplementary figures and images for: Atomoxetine on neurogenic orthostatic hypotension: a randomized, double-blind, placebo-controlled crossover trial
Source: Clin Auton Res. 2024 Sep 19;34(6):561–9. doi: 10.1007/s10286-024-01051-2 (PMC11543771; doi:10.1007/s10286-024-01051-2)

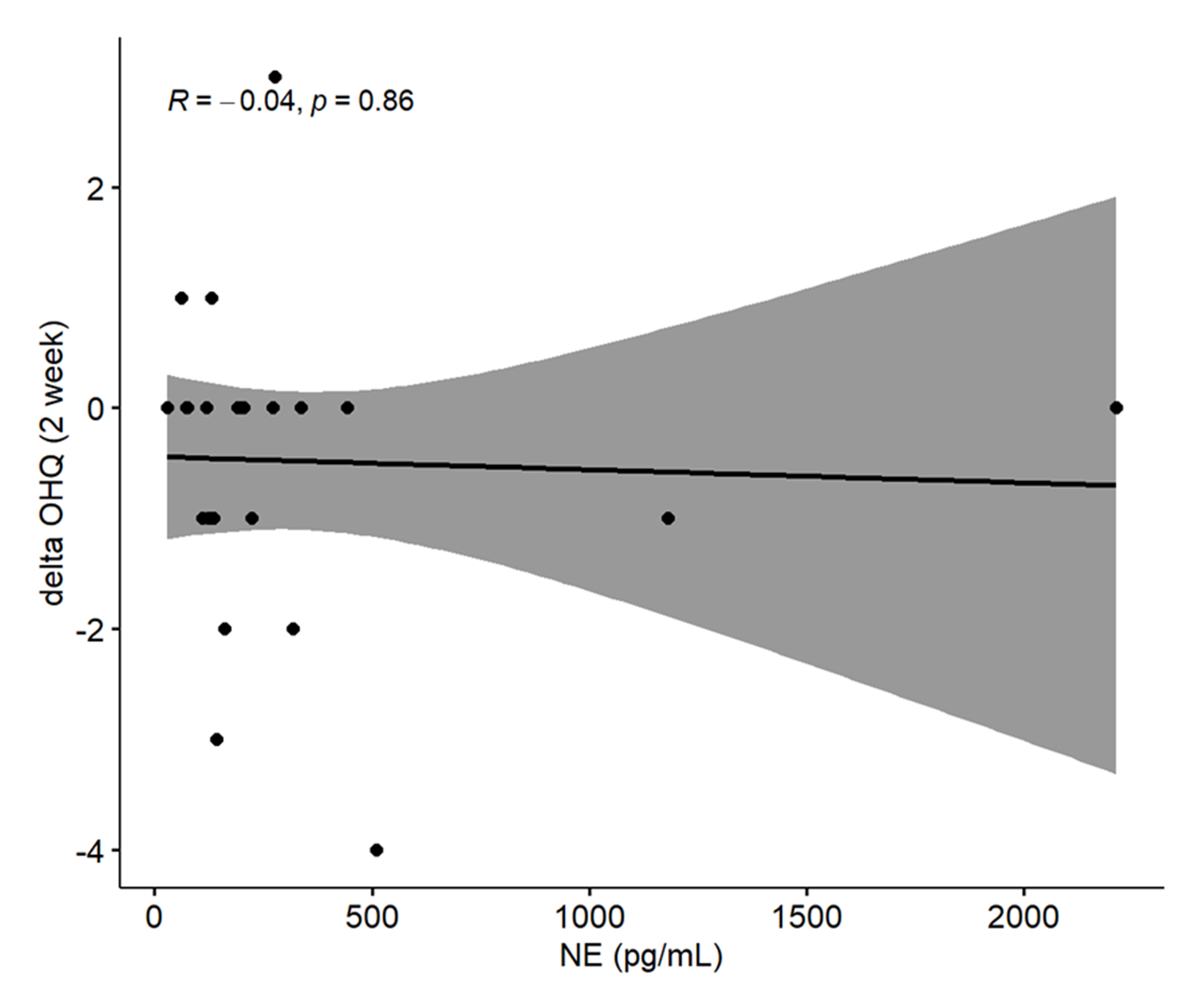

Supplement: Supplementary file 1 — Linear regression analysis on the atomoxetine group. The relationship is not linear. Results of the Shapiro–Wilk test (P < 0.05) and Q–Q plots show none of the norepinephrine supine 15 min, dif_ohq_comp_score_2w, and dif_ohq_comp_score_4w in a normal distribution (TIF 1887 KB) [file 10286_2024_1051_MOESM1_ESM.tif]
