# Supplementary material for: Atomoxetine on neurogenic orthostatic hypotension: a randomized, double-blind, placebo-controlled crossover trial
Source: Clin Auton Res. 2024 Sep 19;34(6):561–9. doi: 10.1007/s10286-024-01051-2 (PMC11543771; doi:10.1007/s10286-024-01051-2)
Supplement: Supplementary file 2 — (DOCX 17 KB) [file 10286_2024_1051_MOESM2_ESM.docx]

| SBP baseline (mean (SD)) | Atomoxetine | Placebo | P-value |
| --- | --- | --- | --- |
| Day 0 | 152.6 (26.5) | 139.7 (21.3) | 0.013 |
| Day 14 | 148.1 (21.5) | 141.8 (21.4) | 0.004 |
| Day 28 | 149.9 (27.2) | 137.3 (23.7) | 0.120 |
| SBP standing 10minutes |  |  |  |
| Day 0 | 104.9 (18.2) | 94.3 (17.2) | 0.044 |
| Day 14 | 99.8 (20.5) | 98.5 (17.3) | 0.103 |
| Day 28 | 102.7 (19.8) | 94.1 (24.6) | 0.103 |

**Table 1. Blood pressure changes on days 0, 14 and 28**

Systolic blood pressure changes at baseline and after standing for ten minutes taken on day 0 which was the first day of drug administration, day 14 and 28.
