# Supplementary material for: Atomoxetine on neurogenic orthostatic hypotension: a randomized, double-blind, placebo-controlled crossover trial
Source: Clin Auton Res. 2024 Sep 19;34(6):561–9. doi: 10.1007/s10286-024-01051-2 (PMC11543771; doi:10.1007/s10286-024-01051-2)
Supplement: Supplementary file 3 — (DOCX 14 KB) [file 10286_2024_1051_MOESM3_ESM.docx]

| Adverse event | Atomoxetine | Placebo |
| --- | --- | --- |
| Headache | 2 | 1 |
| Intermittent goosebumps | 6 | 0 |
| Insomnia | 1 | 1 |
| UTI | 3 | 0 |
| Freezing | 0 | 2 |
| Sore throat and cough | 3 | 0 |
| Fatigue | 0 | 1 |
| Prostatitis | 2 | 0 |
| Constipation | 0 | 2 |

**Table 2. Adverse events**

The ten most encountered adverse events
