# Supplementary material for: Atomoxetine on neurogenic orthostatic hypotension: a randomized, double-blind, placebo-controlled crossover trial
Source: Clin Auton Res. 2024 Sep 19;34(6):561–9. doi: 10.1007/s10286-024-01051-2 (PMC11543771; doi:10.1007/s10286-024-01051-2)
Supplement: Supplementary file 4 — (DOCX 20 KB) [file 10286_2024_1051_MOESM4_ESM.docx]

|  | Placebo | Atomoxetine |  |
| --- | --- | --- | --- |
|  | Mean (SD) | Mean (SD) | P-value |
| **Dizzy** |  |  |  |
| dizzy OHSA baseline | 5.8 (2.4) | 5.3(2.5) | 0.203 |
| dizzy OHSA 2 weeks | 4.8 (2.7) | 4.0 (2.4) | 0.091 |
| dizzy OHSA 4 weeks | 3.9 (2.5) | 3.8 (2.7) | 1.000 |
| **Vision** |  |  |  |
| vision OHSA baseline | 2.8 (2.7) | 2.2 (2.9) | 0.156 |
| vision OHSA 2 weeks | 2.3 (2.6) | 1.6 (2.4) | 0.078 |
| vision OHSA 4 weeks | 2.5 (2.8) | 1.9 (2.2) | 0.755 |
| **Weakness** |  |  |  |
| weakness OHSA baseline | 4.6 (3.2) | 4.4 (2.7) | 0.547 |
| weakness OHSA 2 weeks | 4.3 (3.1) | 3.4 (2.7) | 0.089 |
| weakness OHSA 4 weeks | 3.5 (2.6) | 3.4 (3.0) | 1.000 |
| **Fatigue** |  |  |  |
| fatigue OHSA baseline | 4.9 (3.5) | 4.7 (2.7) | 0.915 |
| fatigue OHSA 2 weeks | 4.9 (2.8) | 3.8 (2.5) | 0.040 |
| fatigue OHSA 4 weeks | 4.1 (3.1) | 3.2 (3.0) | 1.000 |
| **Concentration** |  |  |  |
| Concentration OHSA baseline | 2.6 (2.8) | 2.3 (2.4) | 0.895 |
| concentration OHSA 2 weeks | 2.3 (2.3) | 1.8 (1.9) | 0.326 |
| concentration OHSA 4 weeks | 1.8 (2.4) | 2.0 (2.0) | 1.000 |
| **Head/Neck discomfort** |  |  |  |
| head discomfort OHSA baseline | 2.2 (2.9) | 2.6 (2.6) | 0.344 |
| head discomfort OHSA 2 weeks | 3.0 (3.2) | 2.2 (2.7) | 0.202 |
| head discomfort OHSA 4 weeks | 2.3 (2.5) | 2.4 (2.8) | 0.755 |

**Table 3. Orthostatic hypotension symptom assessment (OHSA)**

Symptoms of orthostatic hypotension where patients grade their symptoms on a scale of 0-10 according to severity.
