# Supplementary material for: Atomoxetine on neurogenic orthostatic hypotension: a randomized, double-blind, placebo-controlled crossover trial
Source: Clin Auton Res. 2024 Sep 19;34(6):561–9. doi: 10.1007/s10286-024-01051-2 (PMC11543771; doi:10.1007/s10286-024-01051-2)
Supplement: Supplementary file 5 — (DOCX 21 KB) [file 10286_2024_1051_MOESM5_ESM.docx]

|  | Placebo | Atomoxetine |  |
| --- | --- | --- | --- |
|  | Mean (SD) | Mean (SD) | P-value |
| **Short stand** |  |  |  |
| Short-stand interference OHDAS baseline | 4.4 (3.1) | 4.6(3.3) | 0.427 |
| Short-stand interference OHDAS 2 weeks | 4.3 (3.2) | 3.2 (2.8) | 0.227 |
| Short-stand interference OHDAS 4 weeks | 2.9 (2.9) | 3.3 (2.9) | 0.135 |
| **Long-stand** |  |  |  |
| Long-stand interference OHDAS baseline | 7.2 (2.7) | 6.3 (3.4) | 0.082 |
| Long-stand interference OHDAS 2 weeks | 6.5 (3.1) | 5.4 (3.8) | 0.058 |
| Long-stand interference OHDAS 4 weeks | 4.8 (3.2) | 5.7 (3.4) | 0.246 |
| **Short walk** |  |  |  |
| Short-walk interference OHDAS baseline | 3.7 (3.0) | 3.4 (3.3) | 0.342 |
| Short-walk interference OHDAS 2 weeks | 3.5 (3.6) | 2.8 (2.8) | 0.557 |
| Short-walk interference OHDAS 4 weeks | 2.8 (2.8) | 2.5 (2.8) | 0.084 |
| **Long walk** |  |  |  |
| Long-walk interference OHDAS baseline | 6.4 (3.2) | 5.3 (3.8) | 0.010 |
| Long-walk interference OHDAS 2 weeks | 6.1 (3.9) | 4.9 (3.9) | 0.145 |
| Long-walk interference OHDAS 4 weeks | 4.9 (3.5) | 5.4 (4.0) | 0.067 |

**Table 4. Orthostatic hypotension daily activity score (OHDAS)**

Patients report their ability to perform daily activities of standing and walking.
